# Polyclonal antibodies inhibit growth of key cellulolytic rumen bacterial species

**DOI:** 10.3389/fmicb.2023.1196492

**Published:** 2023-06-20

**Authors:** Sara M. Tondini, Roderick I. Mackie, Joshua C. McCann

**Affiliations:** ^1^Department of Animal Sciences, University of Illinois at Urbana-Champaign, Urbana, IL, United States; ^2^Carle R. Woese Institute for Genomic Biology, University of Illinois at Urbana-Champaign, Urbana, IL, United States

**Keywords:** rumen fermentation, cellulolytic bacteria, *Fibrobacter succinogenes*, polyclonal antibodies, *Ruminococcus albus*

## Abstract

Antibodies targeting specific bacterial species could allow for modification of the rumen microbial population to enhance rumen fermentation. However, there is limited knowledge of targeted antibody effects on rumen bacteria. Therefore, our objective was to develop efficacious polyclonal antibodies to inhibit the growth of targeted cellulolytic bacteria from the rumen. Egg-derived, polyclonal antibodies were developed against pure cultures of *Ruminococcus albus* 7 (**anti-RA7**), *Ruminococcus albus* 8 (**anti-RA8**), and *Fibrobacter succinogenes* S85 (**anti-FS85**). Antibodies were added to a cellobiose-containing growth medium for each of the three targeted species. Antibody efficacy was determined via inoculation time (0 h and 4 h) and dose response. Antibody doses included: 0 (**CON**), 1.3 × 10^−4^ (**LO**), 0.013 (**MD**), and 1.3 (**HI**) mg antibody per ml of medium. Each targeted species inoculated at 0 h with HI of their respective antibody had decreased (*P* < 0.01) final optical density and total acetate concentration after a 52 h growth period when compared with CON or LO. Live/dead stains of *R. albus* 7 and *F. succinogenes* S85 dosed at 0 h with HI of their respective antibody indicated a decrease (≥ 96%; *P* < 0.05) in live bacterial cells during the mid-log phase compared with CON or LO. Addition of HI of anti-FS85 at 0 h in *F. succinogenes* S85 cultures reduced (*P* < 0.01) total substrate disappearance over 52 h by at least 48% when compared with CON or LO. Cross-reactivity was assessed by adding HI at 0 h to non-targeted bacterial species. Addition of anti-RA8 or anti-RA7 to *F. succinogenes* S85 cultures did not affect (*P* ≥ 0.45) total acetate accumulation after 52 h incubation, indicating that antibodies have less of an inhibitory effect on non-target strains. Addition of anti-FS85 to non-cellulolytic strains did not affect (*P* ≥ 0.89) OD, substrate disappearance, or total VFA concentrations, providing further evidence of specificity against fiber-degrading bacteria. Western blotting with anti-FS85 indicated selective binding to *F. succinogenes* S85 proteins. Identification by LC-MS/MS of 8 selected protein spots indicated 7 were outer membrane proteins. Overall, polyclonal antibodies were more efficacious at inhibiting the growth of targeted cellulolytic bacteria than non-targeted bacteria. Validated polyclonal antibodies could serve as an effective approach to modify rumen bacterial populations.

## 1. Introduction

The rumen ecosystem contains a consortium of microbes that collectively degrade plant biomass and enable ruminants to convert indigestible plant material into digestible food products for human consumption. Feed additives are an important nutritional tool that can modify rumen microbial populations to enhance substrate degradation and rumen fermentation. Among these additives, ionophores and antibiotics can be used to inhibit specific rumen bacteria resulting in improved feed efficiency and animal performance (Callaway et al., 2003). However, the use of antibiotics in beef and dairy production has raised concerns about the emergence of antibiotic-resistant microorganisms (Sharma et al., 2018).

An alternative approach to modulating the rumen microbial population is the application of targeted avian-derived immunoglobulin (IgY). Immunizing laying hens against a target antigen such as bacteria or viruses produces polyclonal egg yolk antibodies that can be easily extracted and administered orally. Unlike monoclonal antibodies, IgY is relatively heat-stable and more resistant to acid digestion and proteolysis (Shimuzu et al., 1988). Oral administration of polyclonal antibodies has been effective against bovine rotavirus and coronavirus, as well as *Salmonella, Staphylococcus*, and *Pseudomonas* (Mine and Kovacs-Nolan, 2002). Polyclonal antibodies generated toward *Streptococcus bovis* and *Fusobacterium necrophorum* dosed in the rumen decreased the abundance of targeted bacteria as measured by most probable number (MPN) viable cell counts (DiLorenzo et al., 2006, 2008) but had varying effects on dry matter intake, volatile fatty acid concentrations, and rumen pH (Marino et al., 2011; Silva et al., 2022a,b). Avian antibodies have also been investigated to reduce methane produced by ruminal archaea (Cook et al., 2008).

The application of this technology could allow for modification of the rumen microbial population to improve fermentation and animal performance. However, the exact mechanisms through which IgY inhibits bacterial activity have not been determined, and there is limited knowledge of targeted IgY application on rumen bacteria in pure culture. A better understanding of dose and time response along with inhibition efficacy and cross-reactivity to non-targeted rumen bacteria strains is needed before polyclonal antibodies can be used as a reliable tool to alter rumen bacterial populations. Cellulolytic bacteria use adherent degradation systems using a variety of cell-surface proteins to utilize substrates in the rumen (Miron et al., 2001). Thus, they serve as suitable model organisms to provide proof-of-concept for polyclonal antibody efficacy as they have many unique target antigens on the cell surface to distinguish mechanisms and extent of cross-reactivity. Therefore, the objective of this study was to develop efficacious polyclonal antibodies and validate their ability to inhibit the growth of targeted cellulolytic bacteria in pure culture.

## 2. Materials and methods

### 2.1. Bacteria

*Ruminococcus albus* 7, *Ruminococcus albus* 8, *Fibrobacter succinogenes* S85*, Streptococcus bovis* JB1*, Prevotella bryantii* B_1_4, and *Megasphaera elsdenii* T81 were obtained from the culture collection of Dr. Roderick Mackie, Department of Animal Sciences at the University of Illinois. Pure cultures were routinely transferred in defined media (Supplementary Table S1), with cellobiose or starch as the sole growth substrate (0.4% w/v). Cells from overnight grown cultures (OD_600_ = 0.8) were used in all growth experiments. Balch tubes containing 9.0 ml of anaerobically prepared medium (Bryant, 1972) were inoculated with 1.0 ml of each respective culture in triplicate.

### 2.2. Antibody preparation

*Ruminococcus albus* 7, *Ruminococcus albus* 8, and *Fibrobacter succinogenes* S85 cells were grown overnight in 10 ml cultures. Cells were inactivated by the addition of 10% formalin and heat treatment at 65°C for 60 min. Cells were pelleted and washed two consecutive times with sodium phosphate-buffered saline (PBS). The washed pellets were suspended in 10 ml of PBS. Polyclonal antibodies were commercially produced (GeneTel Laboratories LLC, Madison, WI). Two hens were immunized with inactivated cells from each bacteria. The first injection was subcutaneous, and the three subsequent injections were intramuscular. All injections included Freund's adjuvant to illicit an immune response. Avian-derived IgY antibodies (13 mg/ml) were, then, extracted from pooled egg yolks (approximately 20 eggs) produced by each hen.

### 2.3. Substrate disappearance

The phenol–sulfuric acid procedure (Wood and Bhat, 1988) was used to determine substrate disappearance. The sample (300 μl) was centrifuged at 20,000 × g for 20 min and mixed with 5% phenol (300 μl) and concentrated sulfuric acid (1.5 ml). Samples were incubated at room temperature for 30 min before reading absorbance at 490 nm. The absorbance values were calculated as glucose equivalents using a standard linear graph (0–100 mg).

### 2.4. Volatile fatty acid assay

Concentrations of volatile fatty acids were determined by gas chromatography using an adapted method by Erwin et al. (1961). In brief, samples were acidified with 2 N HCl and centrifuged at 20,000 × g for 20 min. The supernatant was divided into two tubes, and 250 μl of 25% m-phosphoric acid solution was added to each. Samples were frozen overnight, thawed, and centrifuged at 20,000 × g for 5 min. The supernatant was transferred to gas chromatography vials and analyzed for acetate concentration (mM) in cellulolytic bacteria growth assays or acetate, butyrate, propionate, valerate, isobutyrate, and isovalerate concentrations in non-cellulolytic bacteria growth assays (mM). Volatile fatty acid concentrations were used to characterize fermentation across all strains. Additional end products (succinate, formate, ethanol, and H_2_) were not measured.

### 2.5. Live/dead stain

To measure the viability of each culture, samples were assayed at 12 h with a live/dead stain (LIVE/DEAD BacLight Bacterial Viability Kit). Samples were prepared for readings on a fluorescence microplate, according to the manufacturer's instructions. In brief, 100 μl of dye mixture was added to 100 μl of bacterial suspension, thoroughly mixed, and incubated in the dark for 15 min. Fluorescence intensity was measured at 530 nm and 630 nm to produce a live/dead ratio. Suspensions containing a range of percentages of live cells (100%, 90%, 50%, 10%, and 0%) were used to prepare the standard curves for each strain (relationship between % live bacteria and live/dead ratio).

### 2.6. Scanning electron microscopy

To observe cell surface morphology, samples were taken at h 3 for scanning electron microscopy imaging (Microscopy Suite, Beckman Institute, University of Illinois). Cells were fixed in 2% paraformaldehyde and 2.5% glutaraldehyde. Fixed cells were washed with 0.1 mol/L of sodium cacodylate buffer for 10 min, dehydrated in an ethanol series, and dried by the critical point method. Cells were, then, affixed to aluminum SEM stubs with double-stick carbon tape and sputter-coated with 7 nm of gold-palladium. Samples were examined and imaged on a Field-Emission Environmental Scanning Electron Microscope (FEI Quanta FEG 450 ESEM).

### 2.7. 2D electrophoresis and Western blot

Two-dimensional electrophoresis was performed according to the carrier ampholine method of isoelectric focusing (Kendrick et al., 2020) by Kendrick Labs, Inc. (Madison, WI). In brief, isoelectric focusing was carried out in a glass tube using 2.0% pH 3-10 Isodalt Servalytes (Serva, Heidelberg, Germany) for 9,600 volt-hrs. After equilibration for 10 min in Buffer 'O' (10% glycerol, 50 mM DTT, 2.3% SDS, and 0.0625 M tris, pH 6.8), each tube gel was sealed to the top of a stacking gel that overlaid a 10% acrylamide slab gel (0.75 mm thick). Sodium dodecyl-sulfate (SDS) gel electrophoresis was carried out for 4 h at 15 mA/gel. The gel was dried between sheets of cellophane paper with the acid at the edge to the left.

After slab gel electrophoresis, the gel for blotting was placed in a transfer buffer and transblotted onto a polyvinylidene fluoride (PVDF) membrane overnight. The PVDF membrane was destained in 100% methanol, rinsed briefly in Tween-Tris-buffered saline (TTBS), and blocked for 2 h in 5% non-fat dried milk (NFDM). The blot was, then, incubated in chicken polyclonal antibody (diluted 1:2,000,000 in 2% NFDM TTBS) overnight and rinsed 3 x 10 min in TTBS. The blot was, then, placed in secondary antibody (KPL anti-Chicken Ig-HRP, LGC SeraCare, United States) (diluted 1:5,000 in 2% NFDM TTBS) for 2 h, rinsed as above, treated with ECL (Thermo Fisher Scientific, United States), and exposed to X-ray film (GE Amersham Hyperfilm ECL).

### 2.8. Mass spectrometry

Liquid chromatography with tandem mass spectrometry (LS-MS/MS) analyses were conducted at the Protein Sciences laboratory of the Roy J. Carver Biotechnology Center, University of Illinois at Urbana-Champaign. Gel spots were first destained with 50% acetonitrile (ACN) and then dehydrated with ACN. Next, the gel spots were rehydrated in 50 mM triethylammonium bicarbonate buffer (TEAB) containing 500 ng of proteomics-grade trypsin (Pierce, Thermo Fisher Scientific, United States). The proteins were digested at 55°C for 30 min using a Discover microwave reactor (CEM Corporation, United States), and the resulting peptides were extracted from the gel pieces with 5% formic acid (FA) in 50% ACN. The peptides were dried and desalted with SDB-XC StageTips prior to LC-MS analysis.

The LC-MS analyses were performed with an UltiMate 3000 rsnLC connected to a Q Exactive HF-X mass spectrometer (Thermo Fisher Scientific, United States). The peptides were separated at a flow rate of 300 nL/min on a 25 cm Acclaim PepMap 100 C18 column (Thermo Fisher Scientific, United States) with a 45-min gradient from 5% B to 50% B, where mobile phase A was 0.1% FA and mobile phase B was 0.1% FA in 80% ACN. The mass spectrometer was operated in the positive mode with full MS scans collected at 120k resolution; the top 15 precursors from each MS1 scan were selected for higher energy collisional dissociation (HCD), and the MS2 scans were acquired at 30k resolution. The raw data were searched with Mascot Distiller v2.8.3.0 and Mascot server v.2.8.2 (Matrix Science) against the NCBI *Fibrobacter succinogenes* S85 proteome (9,320 entries; downloaded on January 2023). Search settings included a maximum of two missed cleavages, a peptide mass tolerance of 10 ppm, a fragment mass tolerance of 0.02 Da, and variable modifications of acetylation on protein N-termini and oxidation of methionine residues. High-scoring peptides corresponded to those above the default significance threshold in MASCOT (*P* < 0.05, peptide score >60). Nucleotide sequences were searched through the National Center for Biotechnology Information (NCBI), to determine similarities among proteins annotated as hypothetical.

### 2.9. Determination of antibody efficacy and cross-reactivity

Growth assays were conducted to test the efficacy of anti-RA7, anti-RA8, and anti-FS85 against cell cultures of *R. albus* 7, *R. albus* 8, and *F. succinogenes* S85. Antibody efficacy was evaluated via an inoculation time (0 h and 4 h) and dose-response. Antibody doses included: 0 (**CON**), 1.3 × 10^−4^ (**LO**), 0.013 (**MD**), and 1.3 (**HI**) mg antibody per 1 ml of medium. Doses were determined by executing log-fold dilutions of starting material (13 mg/ml). Inoculation time was evaluated to determine the efficacy of each antibody dose at different cell abundances and growth rates. To test for antibody cross-reactivity, the highest antibody dose (1.3 mg/ml) of all three antibody treatments was added to non-target cultures of each strain at 0 h. Growth was determined by optical density (600 nm) measurements over 52 h. Samples were collected at 6, 12, 18, 24, and 52 h for determination of substrate disappearance and at 12, 24, and 52 h for determination of volatile fatty acid concentration. Samples were collected at 12 h for the live/dead stain assay. Samples were collected at 3 h for SEM imaging.

Additional growth assays were conducted to test for cross-reactivity of anti-FS85 antibodies against cell cultures of the non-target strains *Streptococcus bovis* JB1, *Prevotella bryantii* B_1_4, and *Megaspahera elsdenii* T81. The highest antibody dose (1.3 mg/ml) of anti-FS85 was added to non-target cultures at 0 h to determine cross-reactivity. Growth was determined by optical density (600 nm) measurements over 24 h. Samples were collected at 4, 8, 12, 16, and 24 h for determination of substrate disappearance and volatile fatty acid concentration. To identify antibody binding-specificity of anti-FS85 to *F. succinogenes* S85, two-dimensional electrophoresis and Western blot analyses were performed by Kendrick Labs, INC. (Madison, WI).

### 2.10. Statistical analysis

The MIXED procedure of SAS 9.4 (SAS Inst. Inc., Cary, NC) was used for all statistical analyses. Repeated measures were used to analyze optical density, substrate disappearance, and VFA with fixed effects of treatment, time, and the interaction of treatment and time. Compound symmetry was used as the covariance structure after consideration of the fit statistics. Tukey's test was used for *post hoc* pair-wise comparisons. Significance was declared at *P* ≤ 0.05, and tendencies were discussed at 0.05 < *P* < 0.10.

## 3. Results

### 3.1. Determination of antibody efficacy on targeted strains

#### 3.1.1. Fibrobacter succinogenes S85

**Figure 1:**
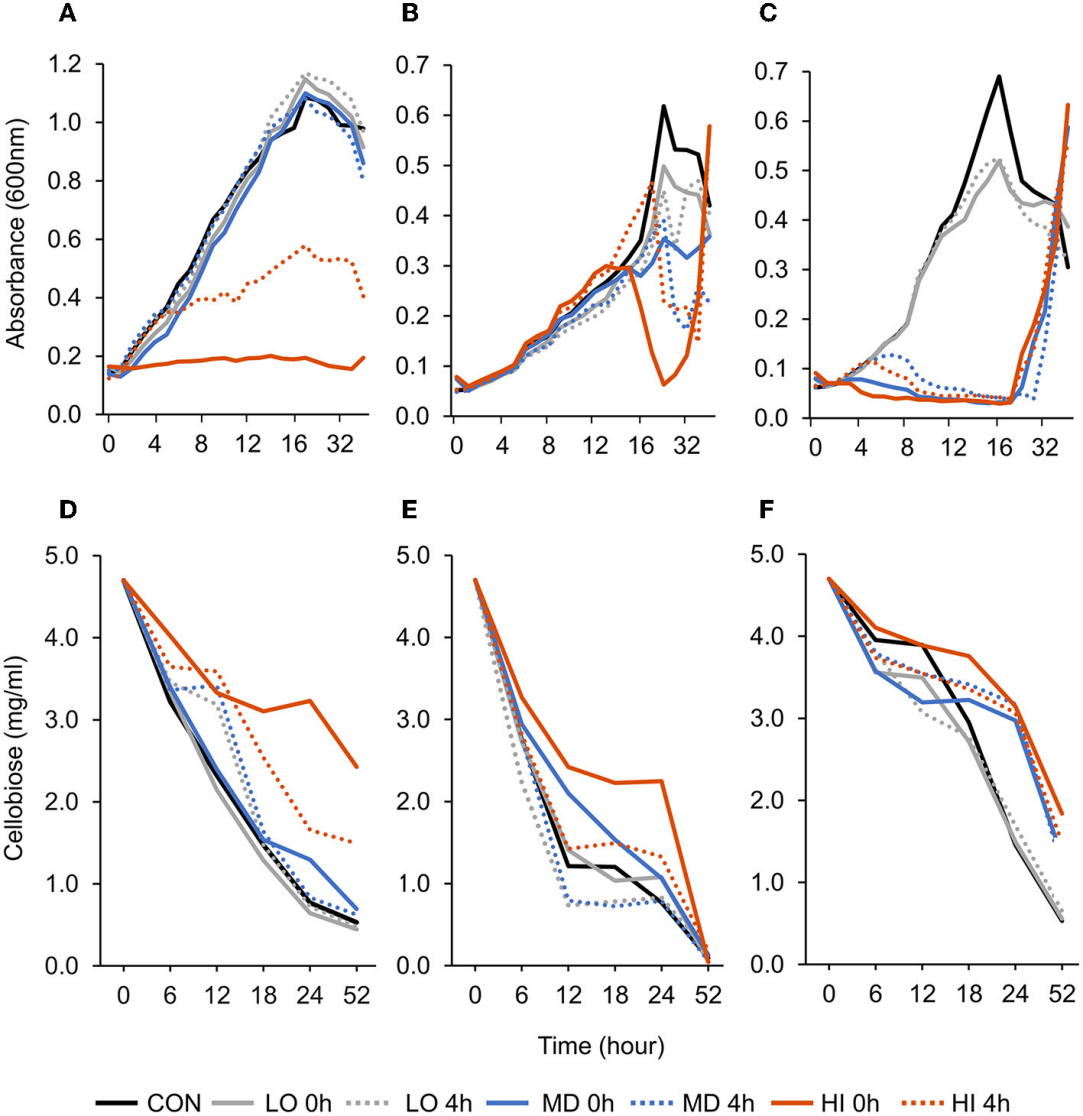
Effect of targeted antibody treatment on optical density and residual cellobiose. **(A–C)** Optical density (600nm). **(A)**—*F. succinogenes*; a treatment × time interaction was observed (*P* < 0.001; SEM = 0.028) **(B)**—*R. albus* 8; a treatment × time interaction was observed (*P* < 0.001; SEM = 0.041). **(C)**—*R. albus* 7; a treatment × time interaction was observed (*P* < 0.001; SEM = 0.038). **(D–F)** Residual cellobiose (mg/ml). **(D)**—*F. succinogenes*; a treatment × time interaction was observed (*P* < 0.001; SEM = 0.136). **(E)**—*R. albus* 8; a tendency for a treatment × time interaction was observed (*P* = 0.09; SEM = 0.275). **(F)**—*R. albus* 7; a treatment × time interaction was observed (*P* < 0.001; SEM = 0.149). Treatment; CON = 0, LO = 1.3 × 10^**–**4^, MD = 0.013, HI = 1.3 mg/ml dosed at 0 h or 4 h.

A treatment × time interaction was observed (*P* < 0.001; Figure 1A) for optical density of *F. succinogenes* S85. Over 9–36 h, addition of HI at 0 h and 4 h decreased OD compared with all other treatments. At 52 h, the addition of HI at 0 h and 4 h decreased OD by 78% and 57%, respectively, when compared with CON. A treatment effect was also observed (*P* < 0.001). Addition of HI at 0 h and 4 h decreased OD compared with CON. Addition of MD at 4 h or LO at 0 h did not affect OD compared with CON. A treatment × time interaction was observed (*P* < 0.001; Figure 1D) for residual cellobiose over 52 h of incubation. The remaining cellobiose at 52 h was greatest in HI at 0 h (2.43 mg/ml), intermediate in HI at 4 h (1.50 mg/ml), and least in all remaining treatments (≤ 0.70 mg/ml).

**Figure 2:**
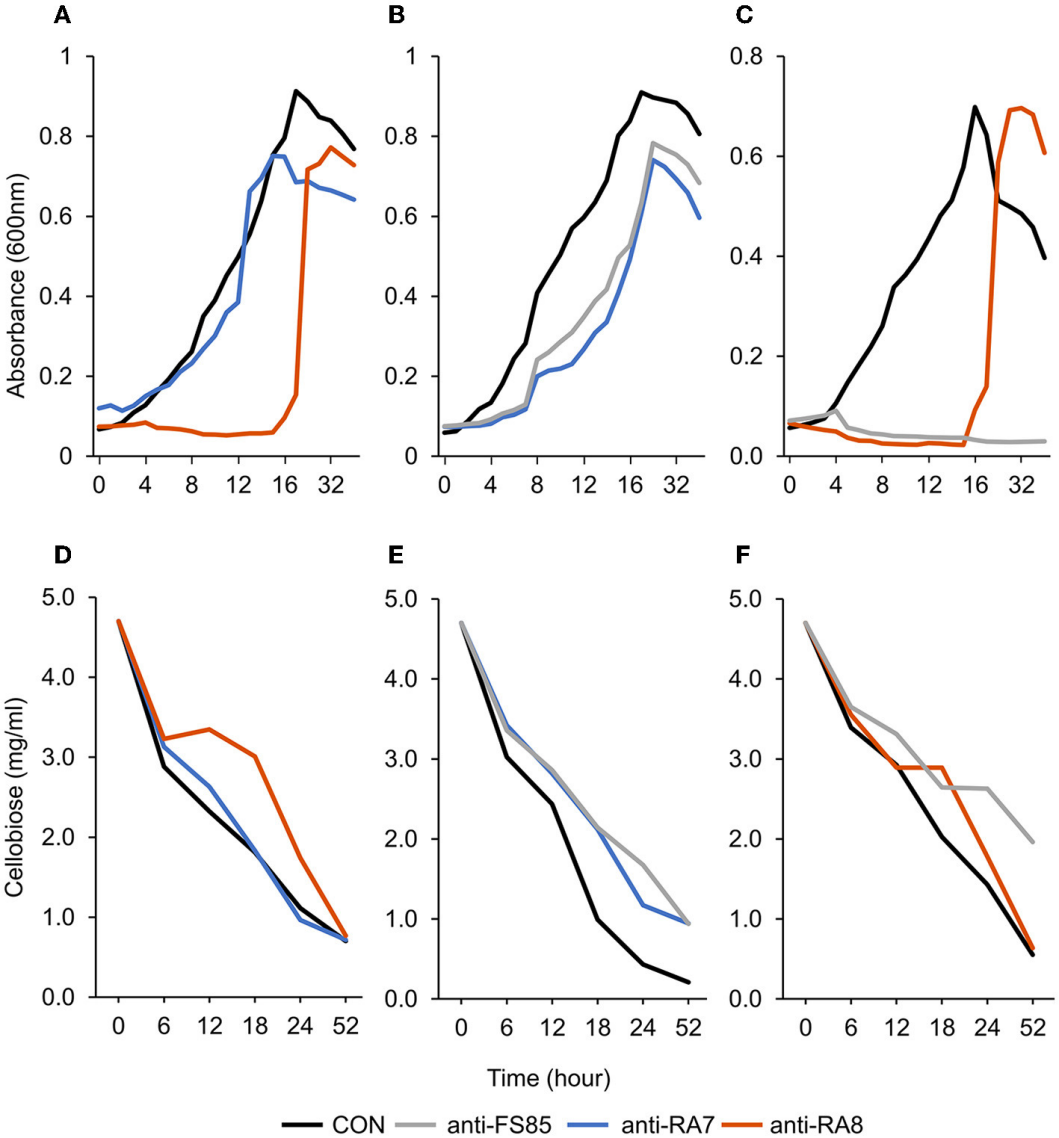
Effect of non-targeted antibody treatment on optical density and residual cellobiose. **(A–C)** Optical density (600 nm). **(A)**—*F. succinogenes*; a treatment × time interaction was observed (*P* < 0.001; SEM = 0.049) **(B)**—*R. albus* 8; a treatment × time interaction was observed (*P* < 0.001; SEM = 0.009). **(C)**—*R. albus* 7; a treatment × time interaction was observed (*P* < 0.001; SEM = 0.008). **(D–F)** Residual cellobiose (mg/ml). **(D)**—*F. succinogenes*; a treatment × time interaction was observed (*P* = 0.003; SEM = 0.138). **(E)**—*R. albus* 8; a treatment × time interaction was observed (*P* = 0.03; SEM = 0.145). **(F)**—*R. albus* 7; a treatment × time interaction was observed (*P* < 0.001; SEM = 0.112). Treatment; CON = 0 mg/ml, anti-FS85 = 1.3 mg/ml of *F. succinogenes* antibody, anti-RA7 = 1.3 mg/ml of *R. albus* 7 antibody, anti-RA8 = 1.3 mg/ml of *R. albus* 8 antibody.

A treatment effect was observed (*P* = 0.01; Figure 2A) for the percentage of live bacterial cells at 12 h. Addition of HI at 0 h decreased live cells by 98% compared with CON. However, CON and addition of LO at 4 h were similar (*P* = 0.99). There were no differences in the percentage of live bacterial cells between LO at 0 h, MD at 0 h and 4 h, and HI at 4 h. Compared with all other treatments, HI at 0 h had the least (*P* < 0.05) amount of live bacterial cells (2.5%). Total acetate concentrations produced by *F. succinogenes* S85 were affected by treatment (*P* = 0.003; Figure 2D). Acetate was greatest in CON (12.4 mM) and least in HI at 0 h (8.5 mM) and 4 h (9.5 mM). Addition of LO or MD at 0 h or 4 h resulted in similar acetate concentrations compared with CON.

#### 3.1.2. Ruminococcus albus 8

A treatment × time interaction was observed (*P* < 0.001; Figure 1B) for optical density of *R. albus* 8. At 24 h, the addition of LO, MD, and HI at 0 h decreased OD by 19, 43, and 90%, respectively, compared with CON. By 52 h, HI at 0 h and 4 h treatments had increased OD compared with CON. Between 36 and 52 h, CON decreased by 20% and HI increased by at least 60%. An overall treatment effect was also observed (*P* < 0.001). Addition of LO at 4 h, MD at 0 h and 4 h, or HI at 0 h and 4 h decreased OD compared with CON or LO at 0 h. Addition of LO, MD, and HI at 4 h decreased OD by 27, 37, and 63%, respectively, compared with CON. A tendency for a treatment × time interaction (*P* = 0.09; Figure 1E) was observed for residual cellobiose over 52 h of incubation. At h 18, HI at 0 h had greater residual cellobiose compared with LO at 0 h and 4 h. At 52 h, all treatments were similar. A treatment effect was also observed (*P* = 0.01). Overall, remaining cellobiose was greatest in HI at 0 h (2.04 mg/ml), least in MD at 4 h (1.02 mg/ml), and LO at 4 h (0.95 mg/ml) and intermediate in all remaining treatments (1.55–1.21 mg/ml).

A treatment effect was observed (*P* = 0.004; Figure 2B) for the percentage of live *R. albus* 8 cells at 12 h. Live percentage of cells was greatest in CON, intermediate in LO, and least in all other treatments. CON and addition of LO at 0 h or 4 h were similar. Addition of MD or HI at 0 h or 4 h decreased live cell percentage compared with CON. Addition of HI at 0 h decreased live cells by 50% compared with CON. No treatment effect (*P* = 0.14; Figure 2E) was observed for the total acetate concentration of *R. albus* 8. Addition of HI, LO, or MD at 0 h or 4 h did not affect acetate production compared with CON.

#### 3.1.3. Ruminococcus albus 7

A treatment × time interaction was observed (*P* < 0.001; Figure 1C) for optical density of *R. albus* 7. At 10–24 h, OD of CON and LO at 0 h or 4 h remained greater compared with all other treatments. At 36 h, all treatments were similar (*P* > 0.99). A treatment effect was also observed (*P* < 0.001) with CON and LO having the greatest OD overall and HI and MD having the least. A treatment × time interaction was observed (*P* < 0.001; Figure 1F) for residual cellobiose over 52 h incubation. Overall, remaining cellobiose was greatest in HI at 0 h (3.35 mg/ml), least in CON (2.56 mg/ml) and LO at 0 h (2.37 mg/ml) and 4 h (2.40 mg/ml), and intermediate in all remaining treatments (3.04–2.86 mg/ml).

A treatment effect was observed (*P* < 0.001; Figure 2C) for the percentage of live *R. albus* 7 cells at 12 h. Addition of MD or HI at 0 h or 4 h decreased live bacterial cell percentage compared with CON or LO at 0 h or 4 h. Addition of MD at 0 h or 4 h decreased cells by 84% and 79%, respectively, compared with CON. Addition of HI at 0 h or 4 h decreased cells by 100% and 99%, respectively, compared with CON. There was a treatment effect (*P* < 0.001; Figure 2F) for *R. albus* 7 acetate concentrations. Addition of MD or HI at 0 h or 4 h decreased acetate concentrations compared with CON or LO at 0 h or 4 h. Addition of HI or MD at 0 h decreased acetate by 19% compared with CON. Addition of HI or MD at 4 h decreased acetate by 19% and 21%, respectively, compared with CON.

### 3.2. Determination of antibody cross-reactivity

#### 3.2.1. Fibrobacter succinogenes S85

**Figure 3:**
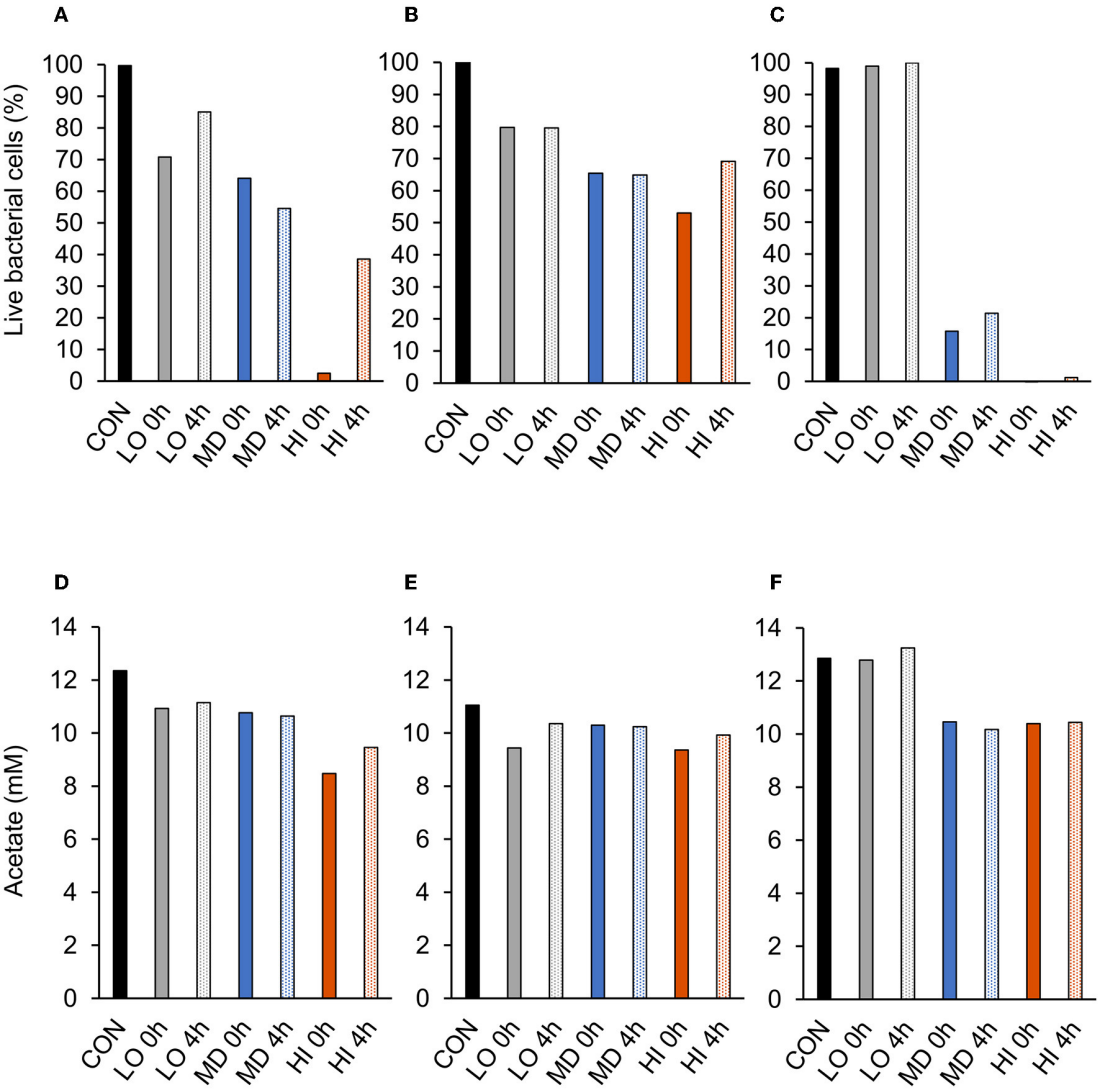
Effect of targeted antibody treatment on live bacterial cells and acetate production. **(A–C)** Live bacterial cells (%) at mid-log phase. **(A)**—*F. succinogenes*; a treatment effect was observed (*P* = 0.01; SEM = 14.64) **(B)**—*R. albus* 8; a treatment effect was observed (*P* = 0.004; SEM = 7.10). **(C)**—*R. albus* 7; a treatment effect was observed (*P* < 0.001; SEM = 5.91). **(D–F)** Total acetate (mM). **(D)**—*F. succinogenes*; a treatment effect was observed (*P* = 0.003; SEM = 0.953). **(E)**—*R. albus* 8; no treatment effect was observed (*P* = 0.14; SEM = 0.656). **(F)**—*R. albus* 7; a treatment effect was observed (*P* < 0.001; SEM = 0.463). Treatment; CON = 0, LO = 1.3 × 10^−4^, MD = 0.013, HI = 1.3 mg/ml dosed at 0 h or 4 h.

A treatment × time interaction was observed (*P* < 0.001; Figure 3A) for optical density of *F. succinogenes* S85. At 24–52 h, treatments were similar. At 11–18 h, the addition of anti-RA8 decreased OD compared with CON and anti-RA7. Additionally, a treatment effect was observed (*P* < 0.001) for optical density of cells with CON being the greatest (0.47 OD), addition of anti-RA7 being intermediate (0.42 OD), and addition of anti-RA8 being the least (0.22 OD). A treatment × time interaction (*P* = 0.003; Figure 3D) was observed for residual cellobiose. At 52 h, the remaining cellobiose was similar (*P* = 1) among treatments. A treatment effect was also observed (*P* < 0.001). Addition of anti-RA8 increased remaining cellobiose compared with anti-RA7 or CON. Addition of anti-RA7 did not affect substrate disappearance in the growth medium over time compared with CON.

**Figure 4:**
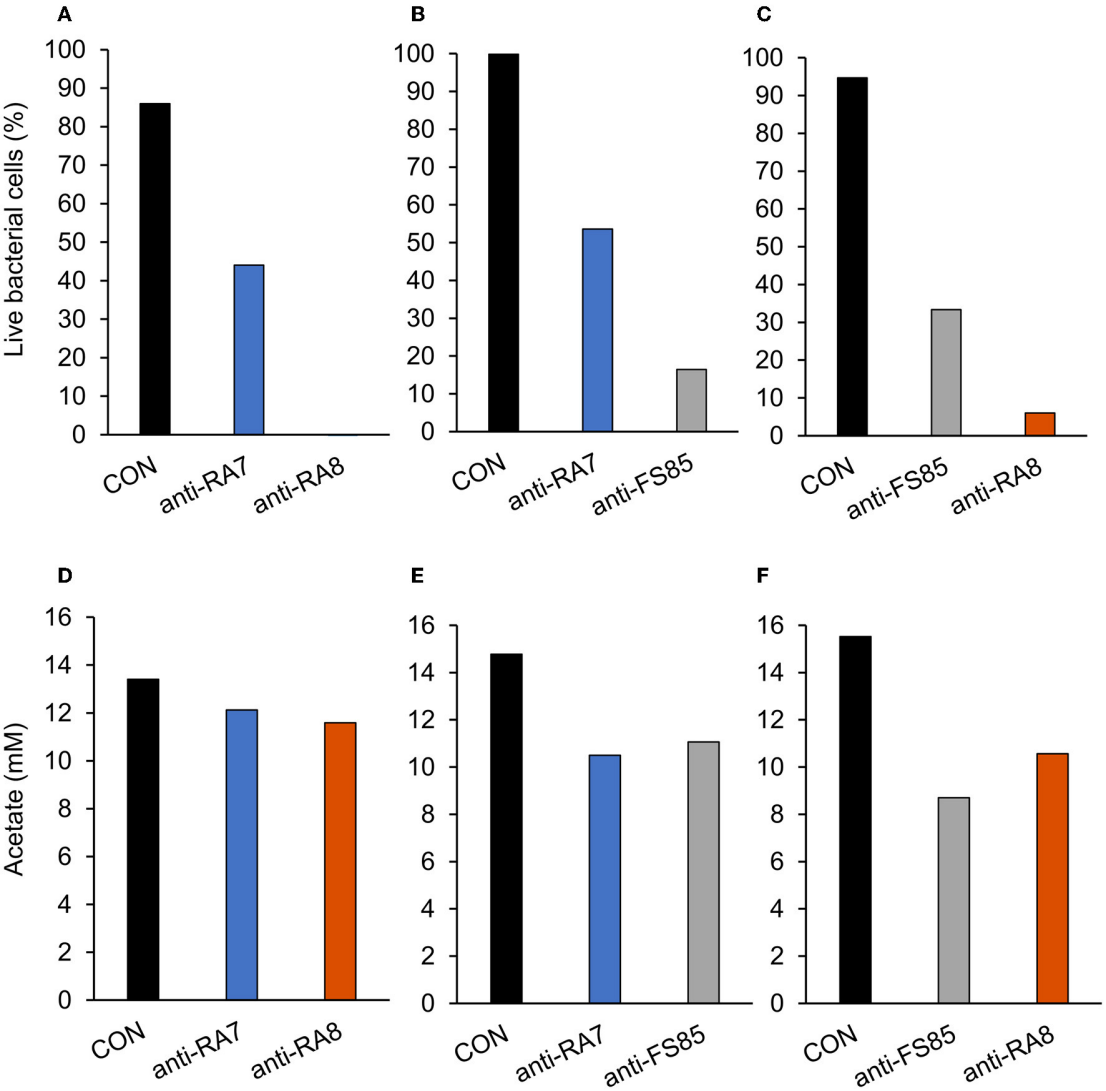
Effect of non-targeted antibody treatment on live bacterial cells and acetate production **(A–C)**; live bacterial cells (%) at mid-log phase. **(A)**—*F. succinogenes*; a treatment effect was observed (*P* < 0.001; SEM = 4.47) **(B)**—*R. albus* 8; a treatment effect was observed (*P* < 0.001; SEM = 5.27). **(C)**—*R. albus* 7; a treatment effect was observed (*P* < 0.001; SEM = 3.89). **(D–F)** R Total acetate (mM). **(D)**—*F. succinogenes*; no treatment effect was observed (*P* = 0.17; SEM = 0.781). **(E)**—*R. albus* 8; a treatment effect was observed (*P* = 0.001; SEM = 0.712). **(F)**—*R. albus* 7; a treatment effect was observed (*P* < 0.001; SEM = 0.574). Treatment; CON = 0 mg/ml, anti-FS85 = 1.3 mg/ml of *F. succinogenes* antibody, anti-RA7 = 1.3 mg/ml of *R. albus* 7 antibody, anti-RA8 = 1.3 mg/ml of *R. albus 8* antibody.

**Figure 5:**
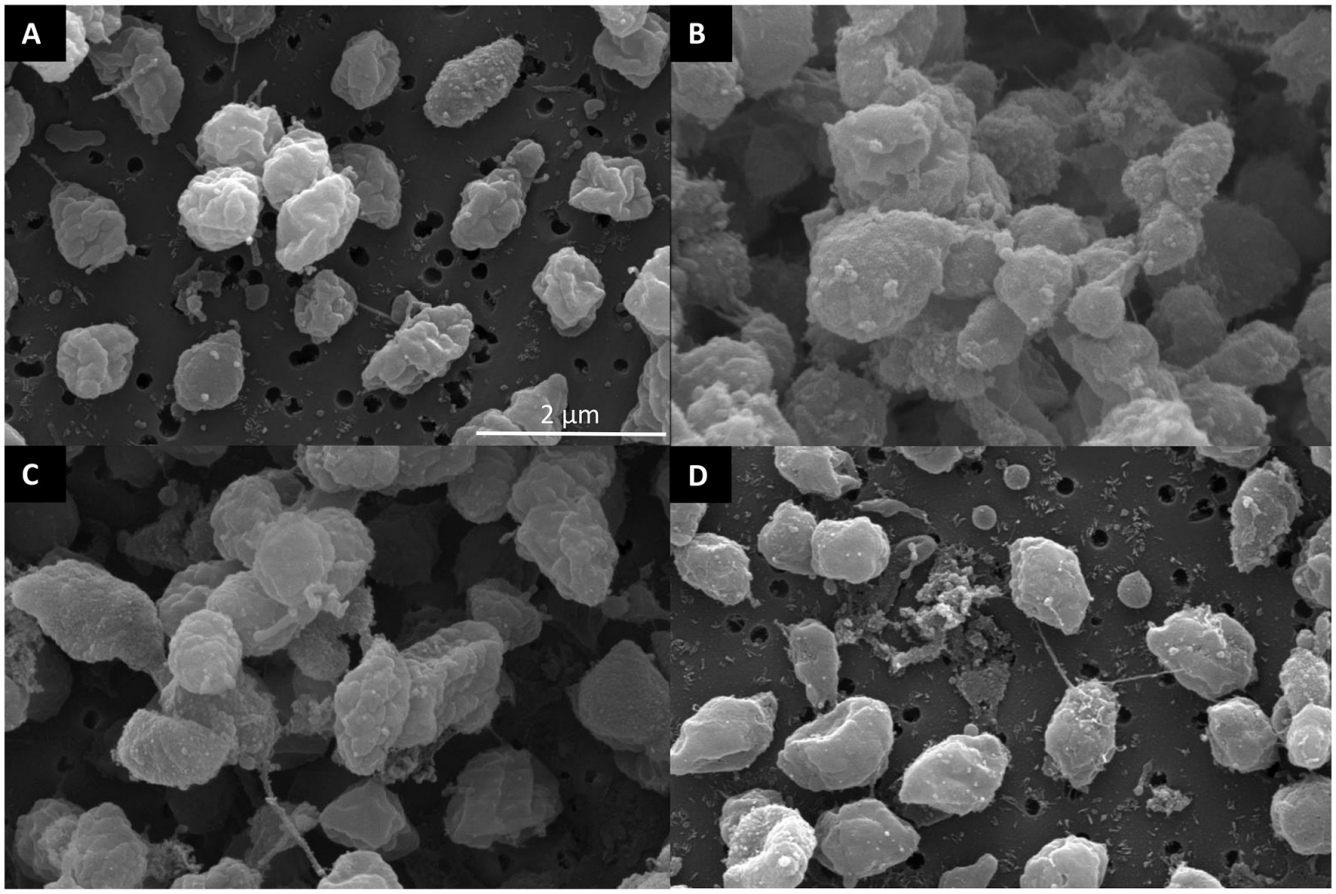
Scanning electron micrographs of surface morphology after antibody treatment of *F. succinogenes* S85. **(A)** CON—no antibody **(B)** 1.3 mg/ml of anti-FS85 **(C)** 1.3 mg/ml of anti-RA7 **(D)** 1.3 mg/ml of anti-RA8.

A treatment effect was observed (*P* < 0.001; Figure 4A) for the percentage of live *F. succinogenes* S85 cells at 12 h with CON being the greatest (86%), anti-RA7 being intermediate (44%), and anti-RA8 being the least (0%). Addition of anti-RA7 decreased live bacterial cells by 49% compared with CON. Addition of anti-RA8 decreased live bacterial cells by 100% compared with CON. No treatment effect (*P* = 0.17; Figure 4D) was observed for total acetate concentrations. Addition of anti-RA7 or anti-RA8 did not affect the overall acetate production compared with CON. Scanning electron microscopy images taken 3 h after the addition of anti-FS85 show more cell-to-cell aggregation and disruption of the cell surface when compared with control cells and the addition of anti-RA7 or anti-RA8 (Figure 5). Extracellular polymeric substance (EPS) was seen in greater amounts on the surface of cells treated with the targeted anti-FS85 compared with the non-targeted antibodies.

#### 3.2.2. Ruminococcus albus 8

A treatment × time interaction was observed (*P* < 0.001; Figure 3B) for optical density of *R. albus* 8. At 0–4 h, treatments were similar. Over 4–9 h, OD decreased in anti-FS85 and anti-RA7 treatments compared with CON. At 10–15 h, the addition of anti-RA7 decreased OD when compared with anti-FS85 or CON. Additionally, a treatment effect was observed (*P* < 0.001) for optical density of cells with CON being the greatest (0.52 OD), anti-FS85 being intermediate (0.37 OD), and anti-RA7 being the least (0.32 OD). A treatment × time interaction (*P* = 0.03; Figure 3E) was observed for residual cellobiose over 52 h incubation. At 6 and 12 h, treatments were similar. At 52 h, there was a tendency (*P* ≤ 0.07) for increased cellobiose remaining in anti-FS85 and anti-RA7 compared with CON. A treatment effect was also observed (*P* < 0.001) with anti-FS85 or anti-RA7 having increased the total remaining cellobiose compared with CON.

A treatment effect was observed (*P* < 0.001; Figure 4B) for the percentage of live bacterial *R. albus* 8 cells at 12 h with CON being the greatest (100%), anti-RA7 being intermediate (54%), and anti-FS85 being the least (16%). Addition of anti-RA7 decreased live bacterial cells by 46% compared with CON. Addition of anti-FS85 decreased live bacterial cells by 84% compared with CON. A treatment effect was observed (*P* = 0.001; Figure 4E) for total *R. albus* 8 acetate concentrations with CON being greater compared with anti-RA7 and anti-FS85. Overall, anti-RA7 decreased acetate by 30% compared with CON. Addition of anti-FS85 decreased acetate by 25% compared with CON. Scanning electron microscopy images taken 3 h after the addition of anti-RA8 show more cell-to-cell aggregation when compared with control and the addition of anti-RA7 or anti-FS85 (Supplementary Figure S1). Cells with anti-RA8 and anti-FS85 show EPS on the surface of cells.

#### 3.2.3. Ruminococcus albus 7

A treatment × time interaction was observed (*P* < 0.001; Figure 3C) for optical density of *R. albus* 7. At 4–18 h, the addition of anti-RA8 decreased OD compared with CON. However, at 24–52 h, the addition of anti-RA8 increased OD compared with CON. Additionally, a treatment effect was observed (*P* < 0.001) for optical density of cells with CON being the greatest (0.35 OD), anti-RA8 being intermediate (0.18 OD), and anti-FS85 being the least (0.05 OD). A treatment × time interaction was observed (*P* < 0.001; Figure 3F) for residual cellobiose over 52 h incubation. At 18 h, the addition of anti-RA8 and anti-FS85 increased the remaining cellobiose compared with CON. At 6, 12, 24, and 52 h, CON and anti-RA8 had similar remaining cellobiose concentrations. At 18 h, the addition of anti-RA8 and anti-FS85 increased the remaining cellobiose by at least 30% when compared with CON. A treatment effect (*P* < 0.001) was observed for residual cellobiose remaining with CON being the greatest (2.07 mg/ml), anti-RA8 being intermediate (2.35 mg/ml), and anti-FS85 being the least (2.84 mg/ml).

A treatment effect was observed (*P* < 0.001; Figure 4C) for the percentage of live *R. albus* 7 cells at 12 h with CON being the greatest (95%), anti-FS85 being intermediate (33%), and anti-RA8 being the least (6%). Addition of anti-FS85 decreased live bacterial cells by 65% when compared with CON. Addition of anti-RA8 decreased live bacterial cells by 94% when compared with CON. A treatment effect (*P* < 0.001; Figure 4F) was observed for total *R. albus* 7 acetate concentrations. Overall, anti-RA8 decreased total acetate by 32% when compared with CON. Addition of anti-FS85 decreased total acetate by 44% compared with CON. Scanning electron microscopy images taken 3h after the addition of anti-RA7 and anti-FS85, show more cell surface EPS compared with the addition of anti-RA8 (Supplementary Figure S2).

### 3.3. Evaluation of anti-FS85 cross-reactivity and antibody-binding specificity

**Figure 6:**
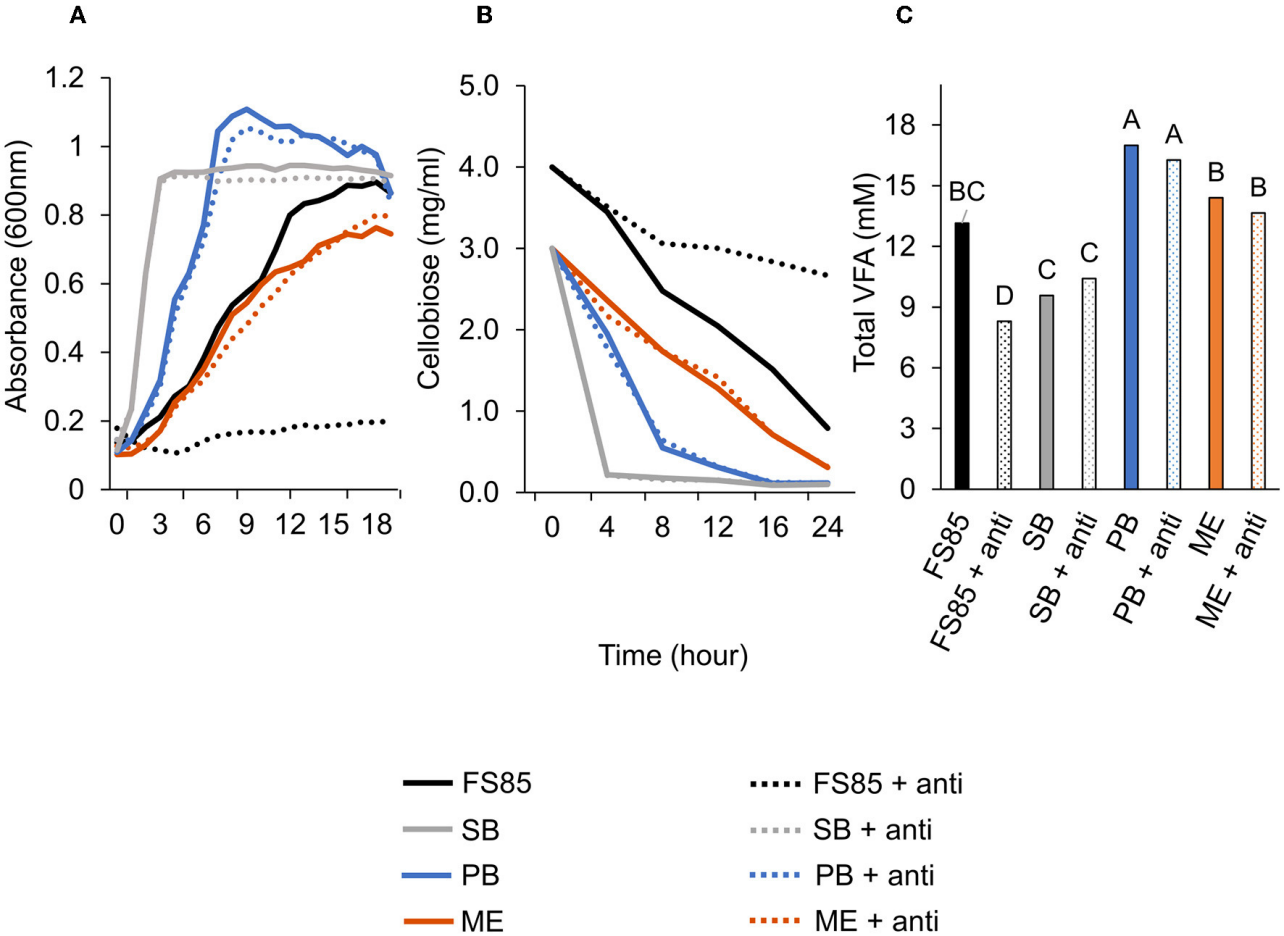
Effect of anti-FS85 antibody treatment on non-cellulolytic bacteria. **(A)**—optical density (600nm) a treatment × time interaction was observed (*P* < 0.001; SEM = 0.012). **(B)**—residual cellobiose (mg/ml) a treatment × time interaction was observed (*P* < 0.001; SEM = 0.070). **(C)**—total volatile fatty acids (mM) a treatment effect was observed (*P* < 0.001; SEM = 0.695). Treatment; FS85 = *F. succinogenes*, SB = *S. bovis* JB1, PB = *P. bryantii* B_1_4, ME = *M. elsdenii* T81, + anti = 1.3mg/ml of anti-FS85. Means with unlike labels differ (*P* ≤ 0.05).

A treatment × time interaction was observed (*P* < 0.001; Figure 6A) for optical densities of *F. succinogenes, S. bovis* JB1, *P. bryantii* B_1_4, and *M. elsdenii* T81. At 3–24 h, addition of anti-FS85 decreased optical density of *F. succinogenes*. However, over 1–24 h, the addition of anti-FS85 to *S. bovis* or *M. elsdenii* did not affect optical density compared with their respective controls. Addition of anti-FS85 to *P. bryantii* decreased optical density at 7 h, but between 8 h and 24 h, optical density was similar to *P. bryantii* control.

A treatment × time interaction was observed (*P* < 0.001; Figure 6B) for residual cellobiose over 24 h incubation. At 8, 12, 16, and 24 h, the addition of anti-FS85 increased the remaining cellobiose in *F. succinogenes* cultures. At 24 h, remaining cellobiose concentrations were similar among *S. bovis* JB1, *M. elsdenii* T81, *P. bryantii* B_1_4, and their respective cultures treated with anti-FS85. A treatment effect was observed (*P* < 0.001; Figure 6C) for total volatile fatty acid concentrations. Addition of anti-FS85 did not affect VFA concentrations in *P. bryantii, S. bovis*, or *M. elsdenii* cultures. However, the addition of anti-FS85 to *F. succinogenes* decreased total VFA concentration by 37%.

**Figure 7:**
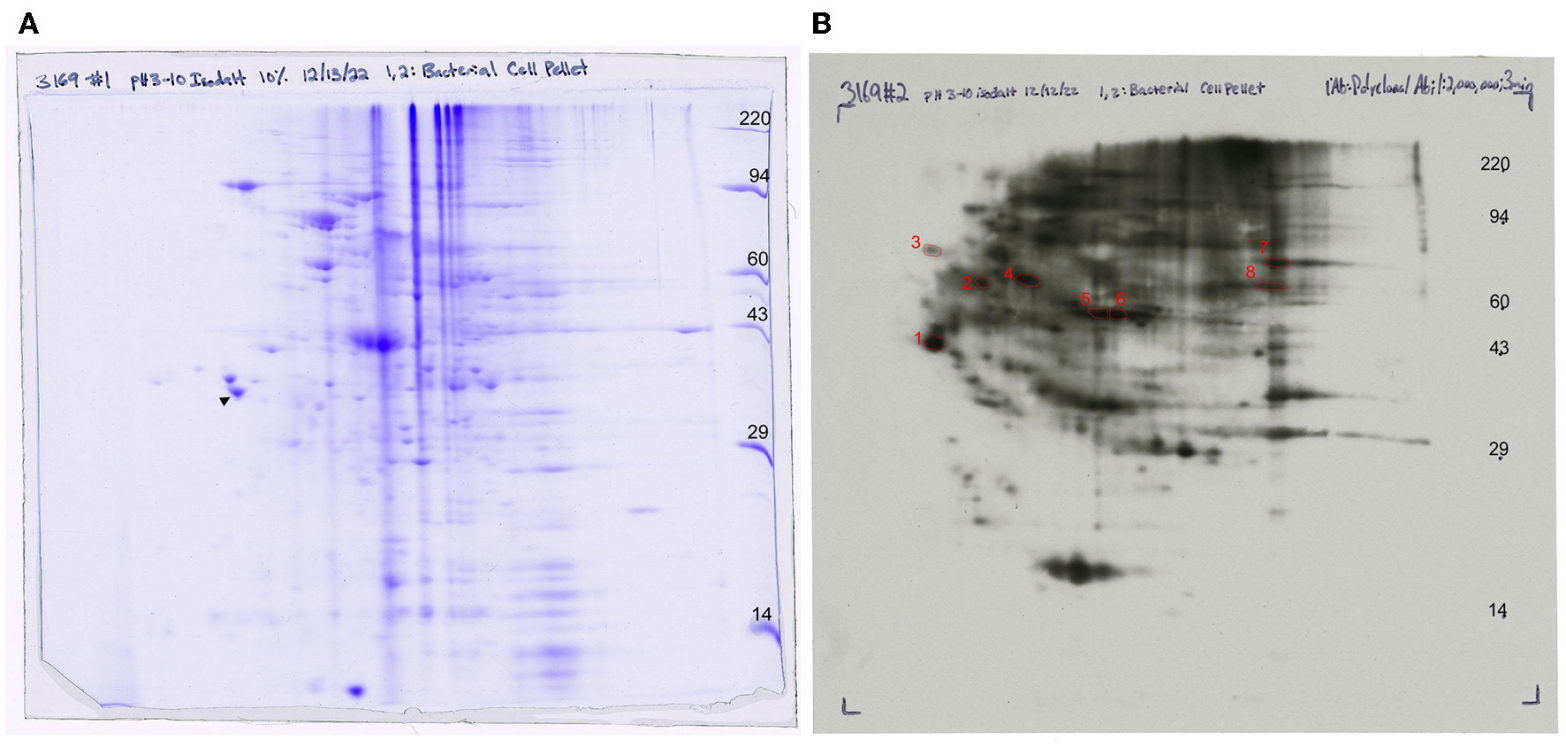
Western blot analysis of anti-FS85 antibody treatment **(A)** 2D SDS PAGE and **(B)** 2D Western blot ECL detection with selected protein spots.

Whole-cell lysate of *F. succinogenes* was subjected to two-dimensional electrophoresis (Figure 7A) to visualize patterns of the cell protein. The final 2D slab gel was transferred to a PVDF membrane, and a Western blot was performed. The spots were visualized on an ECL film after 3 min of X-ray exposure (Figure 7B). Protein patterns on the 2D gel differ from the protein patterns visualized on the ECL film suggesting selective antibody binding.

A total of 10 protein spots were identified on the 2D gel and selected for LC-MS/MS analysis. In total, 9 of the 10 proteins had Mascot scores > 60 (Table 1), indicating successful protein identification. Proteins 1–8 were visualized on ECL film and selected to determine a subset of proteins with anti-FS85 binding-specificity. Proteins 9 and 10 were selected as controls to determine the profile of proteins that were not bound by anti-FS85. Of the eight proteins that bound to anti-FS85, seven were identified as outer membrane proteins. Control protein spots 9 and 10 were identified as cytoplasmic proteins.

**Table 1:** Proteins identified by LC-MS/MS from 2D gel spots.

**Protein #**	**Gene ID^a^**	**Mascot score^b^**	**emPAI^c^**	**Annotation**
1	ADL26237.1	90	0.3	Hypothetical protein FSU_1693
2	ADL24646.1	36	0.07	Conserved domain protein
3	ADL26485.1	85	0.06	Segregation and condensation protein A
4	ADL26888.1	196	0.47	Putative lipoprotein
5	ADL25872.1	1182	4.28	Putative carboxyl-terminal protease
6	ADL24825.1	510	2.22	Outer membrane efflux protein
7	ADL25985.1	874	3.17	Hypothetical protein FSU_0659
8	ADL27203.1	5828	320.98	Translation elongation factor Ts
9	ADL27311.1	397	0.56	Putative saccharopine dehydrogenase
10	ADL25838.1	3019	34.16	Glyceraldehyde 3-phosphate dehydrogenase A

a National Center for Biotechnology Information (NCBI). Bethesda, MD.

b Reflects combined scores of all observed mass spectra that can be matched to amino acid sequence within that protein (Matrix Science).

c 10^PAI^−1, where PAI (Protein Abundance Index) denotes the ratio of observed to observable peptides (Ishihama et al., 2005).

## 4. Discussion

### 4.1. Determination of antibody efficacy on targeted strain

The use of egg-derived, targeted polyclonal antibodies has been shown to inhibit a variety of intestinal pathogens such as bovine and human rotaviruses, bovine coronavirus, *Salmonella, Staphylococcus*, and *Pseudomonas* (Mine and Kovacs-Nolan, 2002) as well as growth of *S. bovis* and *F. necrophorum* in the rumen (DiLorenzo et al., 2006). Additionally, targeted polyclonal antibodies used *in vitro*, inhibited the growth of lipolytic bacteria (Edwards et al., 2017) and several pathogenic bacteria such as *Escherichia coli, Salmonella enteritidis*, and *Helicobacter pylori* (Lee et al., 2002; Sunwoo et al., 2002; Solhi et al., 2017). However, there is limited knowledge of targeted IgY application on rumen bacteria, and a targeted rumen antibody approach has not been validated for efficacy and cross-reactivity in pure culture. The ability to inhibit the growth of specific rumen bacteria could be used to modulate the rumen toward enhanced fermentation patterns.

In the present study, the addition of the highest dose (HI; 1.3 mg/ml) of each antibody into cultures of their targeted strain had the greatest inhibitory effect on OD, substrate utilization, and live bacterial cell percentage when compared with the intermediate (MD; 0.13 mg/ml) or low (LO; 1.3 × 10^−4^ mg/ml) doses. Addition of the HI dose at 0 h decreased total optical density by 74% for *F. succinogenes* S85, 30% for *R. albus* 8, and 68% for *R. albus* 7. The inhibitory effect of HI and MD in *R. albus* 7 persisted for 32 h before those treatments began to increase in OD. This suggests that the mechanism for inhibition may be limited to the initial growth phase when cell counts are low. Bacterial agglutination is a potential mechanism that could reduce OD measurements but not viable cell counts (Tsubokura et al., 2003). Other *in vitro* studies have suggested IgY can cause an adherence blockade that may impair growth-related functions (Jin et al., 1998; Lee et al., 2002). Addition of anti-RA8 at all doses had no effect on growth until 18 h. However, OD of *R. albus* 8 CON remained below 0.35 until 18 h, suggesting slow cell growth may have decreased the opportunity to observe a response to antibody inclusion.

Substrate disappearance was measured to determine the amount of cellobiose remaining throughout the 52 h incubation period. The medium contained 4 mg/ml of cellobiose at the beginning of the experiment. The total remaining cellobiose was greatest (≥ 2.04 mg/ml) with the addition of HI at 0 h. At the end of the incubation period, the addition of HI at 0 h or 4 h resulted in increased remaining cellobiose in *F. succinogenes* S85 and *R. albus* 7 compared with CON. This decreased substrate utilization corresponds with decreased growth measured by OD. In contrast, at 52 h, *R. albus* 8 treatments did not differ and had depleted cellobiose (≤ 0.18 mg/ml). At 24 h, 2.25 mg/ml of cellobiose remained in HI at 0 h in *R. albus* 8-treated cells, indicating most of the substrate utilization for this treatment occurred between 24 h and 52 h. This data also correspond with OD as the addition of HI at 0 h, increased from 0.06 to 0.58 OD between 24 h and 52 h.

Addition of the HI dose (1.3 mg/ml) at 0 h decreased live bacterial cells by 98% for *F. succinogenes* S85, 50% for *R. albus* 8, and 100% for *R. albus* 7. In a similar study, the use of a lower dose (0.5 mg/ml) of egg-derived *Propionibacterium avidum* antibody reduced the lipolytic activity of target *P. avidum* by 59% (Edwards et al., 2017). Addition of 2.5 ml of an *S. bovis*-targeted polyclonal antibody to the feed of ruminally cannulated steers decreased (*P* < 0.05) ruminal MPN enumeration counts of *S. bovis* by 80% (DiLorenzo et al., 2006). Overall, these data suggest that anti-FS85 and anti-RA7 have a greater inhibitory effect on the growth of their targeted strain compared with anti-RA8. However, 1.3 mg/ml of antibody added at 0 h will decrease the growth of each targeted strain over a 52-h incubation period.

### 4.2. Determination of antibody cross-reactivity

Whole-cell preparations of polyclonal antibodies can recognize and bind to many different epitopes of a single antigen. Therefore, cross-reactivity is more likely to occur with polyclonal antibodies than with monoclonal antibodies, which detect a single epitope on the antigen (Frank, 2002). In the present study, antibodies were capable of reducing the growth activity of strains that they were not specifically generated against.

In *F. succinogenes* S85, optical density decreased by 11% in anti-RA7 compared with 54% in anti-RA8 treatments. However, OD was similar in all treatments at 24–52 h. Therefore, *F. succinogenes* S85 cells treated with non-targeted antibodies had greater max OD (0.68) compared with cells treated with targeted antibodies (0.20), suggesting targeted antibodies have greater inhibition efficacy than non-targeted antibodies. At 12 h, live bacterial cells were decreased by 49% with the addition of anti-RA7 and by 100% with the addition of anti-RA8. This data corresponds with the OD at 12 h with CON being the greatest (0.50), anti-RA7 being intermediate (0.40), and anti-RA8 being the least (0.10). However, by 18 h, anti-RA8-treated cells were similar compared with CON, indicating inhibitory effects had decreased by this stage. A similar cross-reactivity response revealed that the addition of 0.5 mg/ml of *P. avidum* polyclonal antibody decreased the lipolytic activity of *P. acnes* by 70% and *B. fibrisolvens* H17c by 49% (Edwards et al., 2017). Scanning electron images show more cell surface disruption with the addition of anti-FS85 compared with anti-RA7 or anti-RA8. Similarly, transmission electron microscopy images of *E. coli* O157:H7 incubated with targeted antibodies resulted in morphological changes and IgY binding around the cell surface (Sunwoo et al., 2002). Overall, these data suggest that the addition of anti-RA8 had a greater inhibitory effect on growth characteristics of *F. succinogenes* S85 than anti-RA7. Additionally, anti-RA7 had lesser inhibitory effects on growth characteristics than HI of anti-FS85.

In *R. albus* 8, optical density decreased by 30% in anti-FS85 and 38% in anti-RA7. Throughout the 52 h incubation period, CON remained the greatest in OD, anti-FS85 was intermediate, and anti-RA7 was the least. The max OD reached by anti-RA7 was 0.74 at 24 h. In experiment 1, the max OD reached by HI at 0 h was 0.58 at 52 h. Substrate disappearance was decreased with the addition of anti-RA7 and anti-FS85 compared with control. However, by 52 h, all treatments had similar remaining cellobiose concentrations. Overall, acetate concentrations were greater in CON compared with anti-FS85 and anti-RA7. At 24 h, anti-FS85 and CON had similar acetate concentrations. These data suggest that both antibodies had an inhibitory growth effect on *R. albus* 8. Similarly, a study aimed to evaluate the inhibitory effects of IgY polyclonal antibodies generated against four strains of *H. pylori* showed a cross-strain inhibitory effect, reducing the growth of non-targeted strains by 29–86% (Solhi et al., 2017).

In *R. albus* 7, optical density decreased by 49% in anti-RA8 and 87% in anti-F85. This percentage of decrease in anti-FS85 is greater than the percentage of decrease in HI at 0 h of anti-RA7 (68%). Interestingly, the anti-RA7 antibody only decreased *F. succinogenes* S85 cells by 11%, suggesting anti-FS85 had a greater inhibitory effect on OD toward *R. albus* 7 than anti-RA7 had on *F. succinogenes* S85. Substrate disappearance was decreased with the addition of anti-RA8 and anti-FS85. At 52 h, CON and anti-RA8 had similar cellobiose remaining. Additionally, live bacterial cell percentage and total acetate were decreased with the addition of anti-RA8 and anti-FS85. Overall, *R. albus* 7 showed the greatest cross-reactivity to non-targeted antibodies with anti-FS85 having a greater inhibitory effect than anti-RA8. Additionally, anti-FS85 and anti-RA7 have similar inhibitory effects on *R. albus* 7 when added at the highest dose. Similar to *H. pylori*, these cellulolytic bacteria may have similar antigens that each antibody could interact with, causing this cross-reactivity effect (Solhi et al., 2017). In contrast, polyclonal antibodies generated against *E.coli* O157:H7 had very little cross-reactivity among other members of the family Enterobacteriaceae (Sunwoo et al., 2002).

### 4.3. Evaluation of anti-FS85 cross-reactivity and antibody-binding specificity

Overall, anti-FS85 displayed greater inhibitory effects on the growth of *F. succinogenes* compared with anti-RA8 and anti-RA7 but was still able to inhibit the growth of the other two cellulolytic strains, *R. albus* 7 and *R. albus* 8. Further evaluation of this antibody was conducted to explore its effects on non-cellulolytic rumen bacteria. *S. bovis* JB1, *P. bryantii* B_1_4, and *M. elsdenii* T81 were dosed with 1.3 mg/ml of anti-FS85. Overall, OD, substrate disappearance, and total VFA were not affected by the addition of anti-FS85. As expected, *F. succinogenes* had decreased OD, decreased VFA concentrations, and increased residual cellobiose when dosed with 1.3 mg/ml of anti-FS85. These results indicate that anti-FS85 has greater binding specificity to *F. succinogenes* when compared with *S. bovis* JB1, *P. bryantii* B_1_4, and *M. elsdenii* T81, suggesting that the epitope regions targeted by anti-FS85 are not located on these non-cellulolytic rumen bacteria strains.

A 2D Western blot was performed to further evaluate antibody-binding specificity. The Western blotting process relies on the primary antibody's specificity, to recognize and bind to its target antigen, and selectivity, to bind to its target antigen in the presence of other proteins (Pillai-Kastoori et al., 2020). As expected, anti-FS85 was able to recognize and bind to multiple epitopes but did not bind to all proteins separated on the 2D gel. This suggests that anti-FS85 has both specificity and selectivity toward its target antigens.

To identify these epitope regions, eight protein spots were excised and analyzed through LC-MS/MS. Protein 1 was annotated as hypothetical protein FSU_0659 and was most likely associated with pilus assembly. This protein shares motifs with type IV pilus assembly proteins and is clustered with genes annotated as pilus assembly proteins (FSU_0658, FSU_0659, FSU_0660, FSU_0661, and FSU_0662). Type IV pili are filaments located on the cell surface of bacteria and have been shown to play a role in cellulose adhesion in *R. albus* and *R. flavefaciens* (Vodovnik et al., 2013). Protein 2 was annotated as a conserved domain protein identified from outer membrane proteins in *F. succinogenes* S85 (Jun et al., 2007). Protein 3 was annotated as FSU_1323, a segregation and condensation protein A that forms part of the condensin complex necessary for chromosomal partition during cell division. The condensin complex pulls DNA away from the mid-cell into both cell halves (Dervyn et al., 2004). Protein 4 was annotated as a putative lipoprotein, which is a hydrophilic protein anchored to the cell membrane (Wilson and Bernstein, 2016). Similarly, *F. succinogenes* outer membrane proteins were identified from cells grown on cellulose and glucose (Jun et al., 2007), and four of the identified proteins were putative lipoproteins. Protein 5 was identified as a putative-carboxyl-terminal protease (CtpA), which functions in post-translational protein processing (Satoh and Yamamoto, 2007). These proteins have been identified on the outer membrane of *Borrelia burgdorferi* (Östberg et al., 2004). Protein 6 was annotated as an outer membrane efflux protein and functions as a channel to allow the export of substrates in Gram-negative bacteria (Johnson and Church, 1999). The most abundant protein, with an emPAI score of 320, was annotated as a translation elongation factor Ts, which functions alongside EF-Tu to assist in protein synthesis (Burnett et al., 2013). EF-Tu has been found on the cell surface of Gram-positive and Gram-negative bacteria (Granato et al., 2004). The control proteins that did not bind to anti-FS85 were annotated as a putative saccharopine dehydrogenase (protein 9) and a glyceraldehyde 3-phosphate dehydrogenase (protein 10). These proteins are located in the cytoplasm. Of the eight selected proteins that did bind to anti-FS85, seven were associated with the outer membrane, and SEM images indicated that anti-FS85 caused cell surface disruption to *F. succinogenes*. This suggests that the anti-FS85 antibody is binding or interacting with cell surface proteins. This may prevent the function of OM proteins or disrupt cellular components through signal transduction, leading to cell death (Wade and O'Toole, 2010). Thus, developing polyclonal antibodies from heat-inactivated whole-cell lysates may be an effective way to target outer membrane proteins of specific bacteria to inhibit growth. Future studies should address antibody-binding specificity toward Gram-positive bacterial cells as they lack an outer membrane layer and toward non-targeted strains that display high levels of cross-reactivity.

## 5. Conclusion

Overall, polyclonal antibodies generated against key cellulolytic rumen bacterial strains inhibited the growth of targeted strains in monoculture. Additionally, antibodies were capable of reducing the growth activity of strains that they were not specifically generated against. However, these inhibitory effects on growth characteristics of non-targeted strains are less impactful to the overall growth of cells over a 52-h incubation period. This suggests that while there is cross-reactivity, there may be a greater specificity for antibodies generated against their target strain. Additionally, polyclonal antibodies generated against whole bacterial cell lysates appear to target outer membrane proteins. This method of antibody generation could be used in future studies to inhibit the growth of specific bacteria to modify rumen microbial populations toward improved fermentation patterns and increased animal performance. Determining the affinity for targeted and non-targeted strains in mixed cultures can further validate antibody selectivity and efficacy.

## Data availability statement

The raw data supporting the conclusions of this article will be made available by the authors, without undue reservation.

## Author contributions

ST contributed to acquisition and analysis of data and wrote the first draft of the manuscript. All authors contributed to the design of the study, manuscript revision, and approved the submitted version.
