# Supplementary material for: Polyclonal antibodies inhibit growth of key cellulolytic rumen bacterial species
Source: Front Microbiol. 2023 Jun 20;14:1196492. doi: 10.3389/fmicb.2023.1196492 (PMC10318403; doi:10.3389/fmicb.2023.1196492)

Supplementary Material

1 Supplementary Tables and Figures

1.1 Supplementary Tables

Supplementary Table 1. Anaerobic medium for culturing cellulolytic bacteria

Ingredient	Concentration in stock (per liter)	Concentration in Media (per liter)
Cellobiose[1]		4 g
Trypticase		2 g
Yeast Extract		1 g
Mineral 1		
K_2HPO_4	6 g	50 ml
Mineral 2		50 ml
KH_2PO_4	6 g	
$(NH_4)_2SO_4$	6 g	
NaCl	12 g	
$MgSO_4 * H_2O$	2.45 g	
$CaCl_2 * 2H_2O$	1.59 g	
VFA Solution		3ml
Acetic acid	13.7 ml	
Propionic acid	6 ml	
Butyric acid	3.68 ml	
Isobutyric acid	1.1 ml	
2-methylbutyric acid	0.94 ml	
n-valeric acid	1.1 ml	
Isovaleric acids	1.1 ml	
Phenylacetate	340 mg	
Phenylpropionate	375 mg	
Resazurin (0.1%)		300 ul
Cysteine HCl		0.6 g
Sodium carbonate		4 g

[1]Starch was used for growth of *S. bovis* JB1, *P. bryantii* B_14, and *M. elsdenii* T81

Supplementary Figure S1: Scanning electron micrographs of surface morphology after antibody treatment of *R. albus 8*. (A) CON – no antibody (B) 1.3 mg/ml of anti-RA8 (C) 1.3 mg/ml of anti-RA7 (D) 1.3 mg/ml of anti-FS85.

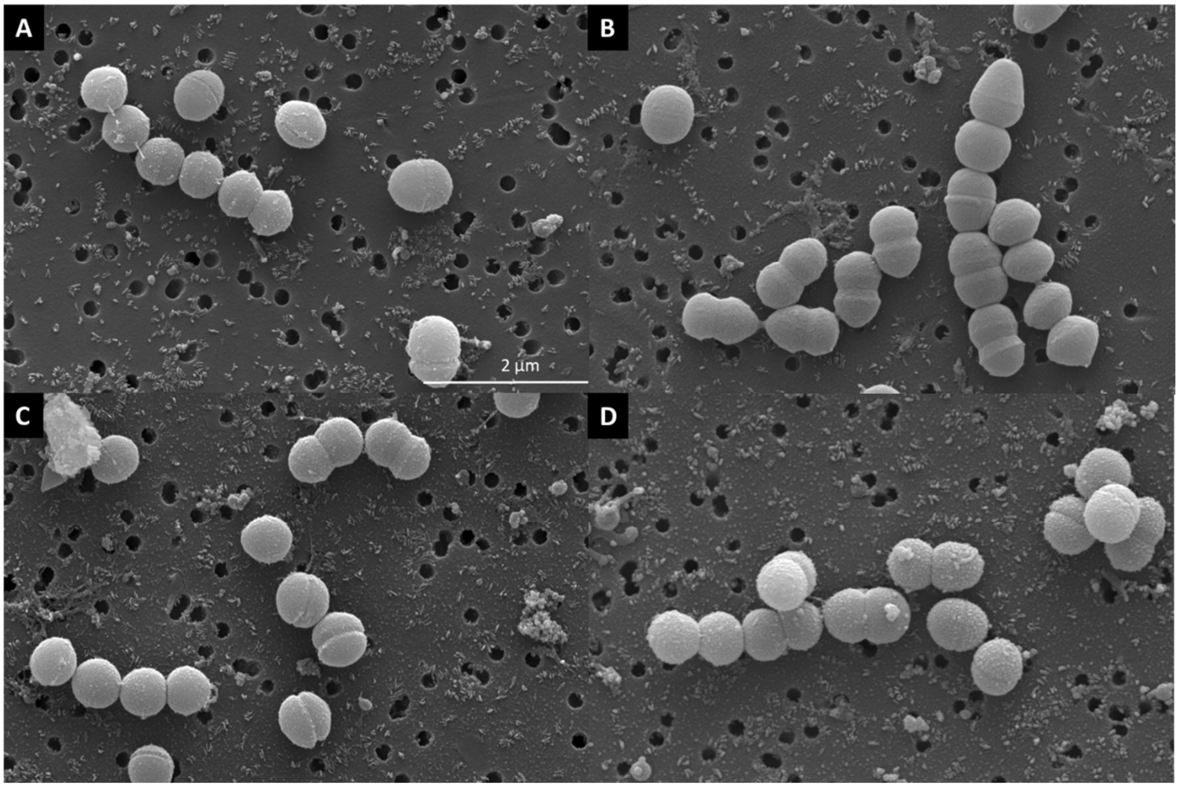

Supplementary Figure S2: Scanning electron micrographs of surface morphology after antibody treatment of *R. albus 7* (A) CON – no antibody (B) 1.3 mg/ml of anti-RA7 (C) 1.3 mg/ml of anti-RA8 (D) 1.3 mg/ml of anti-FS85.

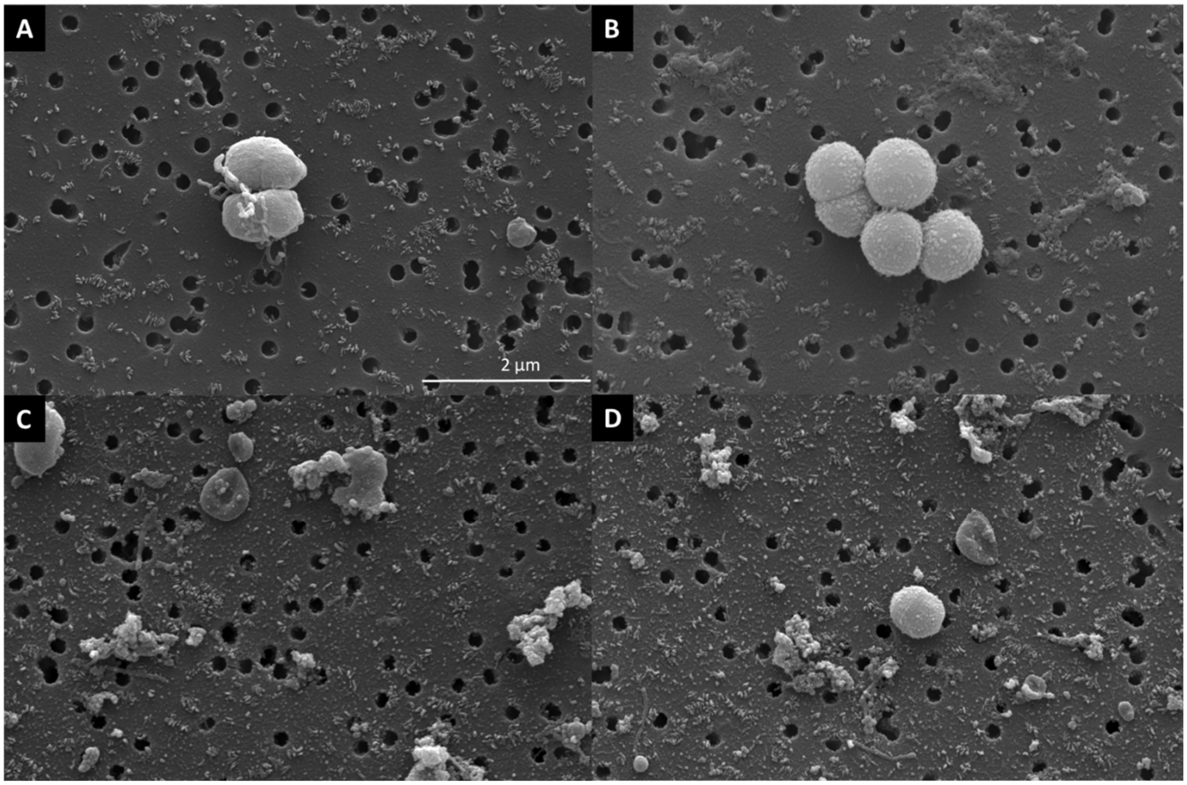

Supplement: Supplementary file 1 [file Data_Sheet_1.pdf]
